# Individual inflammatory marker abnormalities or inflammatory marker scores to identify primary care patients with unexpected weight loss for cancer investigation?

**DOI:** 10.1038/s41416-021-01282-4

**Published:** 2021-02-09

**Authors:** B. D. Nicholson, J. L. Oke, P. Aveyard, W. T. Hamilton, F. D. R. Hobbs

**Affiliations:** 1 Nuffield Department of Primary Care Health Sciences, University of Oxford, Oxford, UK; 2 Medical School, University of Exeter, Exeter, UK

**Keywords:** Cancer epidemiology, Digestive signs and symptoms, Diagnostic markers

## Abstract

**Background:**

Combinations of inflammatory markers are used as prognostic scores in cancer patients with cachexia. We investigated whether they could also be used to prioritise patients attending primary care with unexpected weight loss for cancer investigation.

**Methods:**

We used English primary care electronic health records data linked to cancer registry data from 12,024 patients with coded unexpected weight loss. For each individual inflammatory marker and score we estimated the sensitivity, specificity, likelihood ratios, positive predictive value (PPV) and the area under the curve along with 95% confidence intervals for a cancer diagnosis within six months.

**Results:**

The risk of cancer associated with two abnormal inflammatory markers combined in a score was higher than the risk associated with individual inflammatory marker abnormalities. However, the risk of cancer in weight loss associated with individual abnormalities, notably a raised C-reactive protein, was sufficient to trigger further investigation for cancer under current NICE guidelines.

**Conclusions:**

If scores including pairs of inflammatory marker abnormalities were to be used, in preference to individual abnormalities, fewer people would be investigated to diagnose one cancer with fewer false positives, but fewer people with cancer would be diagnosed overall.

## Background

The evidence base for using simple blood tests to identify symptomatic adults attending primary care at increased risk of cancer is almost entirely limited to tests used in isolation.^1^ With the exception of anaemia (for colorectal cancer) and jaundice (for pancreatic cancer) the risk of individual cancers associated with single blood test abnormalities is below the three percent threshold recommended by NICE for urgent cancer investigation.^1^ Thrombocytosis, hypercalcaemia and hypoalbuminaemia are associated with increased cancer risk across multiple sites.^1^ Combinations of abnormal tests may hold greater potential to rule-in and rule-out further cancer investigation but research is lacking on the most predictive combinations in symptomatic patients.^2,3^

Inflammatory marker scores are used by hospital specialists as surrogate markers of the catabolic drive in cancer cachexia.^4^ Raised scores correlate with the extent of weight loss (WL), predict symptom burden, tumour progression and survival across multiple cancer types.^5^ Commonly reported scores are the modified Glasgow prognostic score (mGPS), that combines low albumin and raised C-reactive protein (CRP), the neutrophil–lymphocyte score (NLS) and the platelet–lymphocyte score (PLS).

Unexpected WL may represent the first clinical manifestation of cancer in primary care. Cancer risk rises above 3% when adults with unexpected WL also have low albumin or a raised white cell count, calcium, platelets or inflammatory markers.^6^ The aim of this study was to investigate whether inflammatory marker scores could be used to select patients with unexpected WL for further cancer investigation in preference to individual inflammatory marker abnormalities.

## Method

We used primary care electronic health records from the Clinical Practice Research Datalink (CPRD), a representative anonymised English database, from between 1 January 2000 to 31 December 2012 and linked to the National Cancer Registrations and Analysis Service (NCRAS) cancer register.^7^ Patients were included if aged ≥18 and registered for at least 12 months with a CPRD general practice before the index date. The index date was the first validated unexpected WL code. These codes equate to a mean weight loss of 5% or more within a 6-month period.^6^ All patients had the following inflammatory markers recorded in the 3 months before to 1 month after the index date: albumin, CRP, lymphocyte count (LC), neutrophil count (NC) and platelet count (PC). We excluded patients with evidence of a past cancer diagnosis or a WL intervention within the previous six months. Cancers coded in the six months after the index date were identified in the CPRD and NCRAS data using an existing library of codes to include all high level ICD-O categories.^8^ Non-melanoma skin, in situ, benign, ill defined or uncertain cancers were not included.

The following thresholds were used for abnormal inflammatory markers: albumin < 35 g/l; CRP > 10 mg/l; LC < 1.5 × 10^9^/l; NC > 7.5 × 10^9^/l; PC > 400 × 10^9^/l. The following inflammatory marker scores were derived: mGPS = 2 (CRP > 10 mg/l and albumin < 35 g/l); NLS = 2 (NC > 7.5 × 10^9^/l and LC < 1.5 × 10^9^/l); and PLS = 2 (PC > 400 × 10^9^/l and LC < 1.5 × 10^9^/l).^4^ For each individual inflammatory marker and score we estimated the sensitivity, specificity, likelihood ratios, positive predictive value (PPV) and the area under the curve along with 95% confidence intervals using the DIAGT Stata module. We present PPVs for age-groups incorporated into clinical guidance.

## Results

Of 63,973 people with a code for unexpected WL, 12,024 (18.8%) had all five inflammatory markers recorded; 5,214 (45.0%) were men, median age 63 years (interquartile range, 47–76 years) and 6612 (55.0%) were women, 66 years (45–79 years). There were 217 (1.80%) people diagnosed with cancer within 6 months, of whom 191 (88.0%) were aged ≥60 years.

The highest AUCs for individual inflammatory markers were for CRP (0.76 (95% CI 0.73–0.79)) and neutrophils (0.64 (0.61–0.67)) (Table 1). Scores with the greatest AUCs were mGPS = 2 (0.64 (0.61–0.67)) and NLS = 2 (0.58 (95% CI 0.55–0.60)). The testing strategy with highest specificity was PLS = 2 (98.65% (98.43–98.85%) followed by NLS = 2 (96.73% (96.39–97.04%)). Raised platelets and all scores had a positive likelihood ratio ≥5.0, the rule of thumb to identify a good rule-in test.^9^ The testing strategy with highest sensitivity was a raised CRP (70.97% (64.44–76.91%)) followed by low lymphocytes (46.54% (95% CI 39.76–53.42%). No normal maker or score had a negative likelihood ratio < 0.2, the rule of thumb to identify a reliable rule-out test.^9^

**Table 1:** Diagnostic accuracy of three inflammatory marker scores for a cancer diagnosis within 6 months of a first presentation to primary care with unexpected weight loss.

	TP	FP	FN	TN	AUC	Sens (%)	Spec (%)	PLR	NLR	PPV (%)			
										≥18 y	40–59 y	60–79 y	≥80 y
Unexpected WL	217	11,807								1.80 (1.57–2.06)	0.78 (0.5–1.16)	***2.65 (2.20–3.16)***	***2.99 (2.35–3.75)***
*Individual inflammatory markers in people with unexpected WL*													
Low albumin	60	790	157	11,017	0.60 (0.57–0.63)	27.65 (21.81–34.11)	93.31 (92.84–93.75)	4.13 (3.30–5.18)	0.78 (0.71–0.84)	**7.06 (5.43–8.99)**	*3.81 (1.05–9.47)*	**10.6 (7.65–14.20)**	*4.94 (2.90–7.79)*
Raised CRP	154	2,306	63	9,501	0.76 (0.73–0.79)	70.97 (64.44–76.91)	80.47 (79.74–81.18)	3.63 (3.31–3.99)	0.36 (0.29–0.44)	**6.26 (5.34–7.29)**	*3.27 (1.75–5.53)*	**7.71 (6.21–9.43)**	**7.01 (5.31–9.05)**
Raised neutrophils	85	1,299	132	10,508	0.64 (0.61–0.67)	39.17 (32.63–46.01)	89.00 (88.42–89.56)	3.56 (2.99–4.24)	0.68 (0.61–0.76)	**6.14 (4.93–7.54)**	*3.02 (1.39–5.66)*	**7.36 (5.38–9.79)**	**9.07 (6.28–12.56)**
Low lymphocytes	101	3,377	116	8,430	0.59 (0.56–0.62)	46.54 (39.76–53.42)	71.40 (70.57–72.21)	1.63 (1.41–1.88)	0.75 (0.66–0.85)	***2.90 (2.37–3.52)***	1.91 (1.02–3.24)	*3.74 (2.80–4.89)*	*3.26 (2.29–4.49)*
Raised platelets	50	534	167	11,273	0.59 (0.56–0.62)	23.04 (17.61–29.22)	95.48 (95.09–95.85)	5.09 (3.94–6.59)	0.81 (0.75–0.87)	**8.56 (6.42–11.13)**	**6.87 (3.19–12.64)**	**9.62 (6.49–13.61)**	**10.08 (5.32–16.95)**
*Inflammatory marker scores in people with unexpected WL*													
mGPS = 2	74	762	143	11,045	0.64 (0.61–0.67)	34.10 (27.82–40.82)	93.55 (93.09–93.98)	5.28 (4.34–6.44)	0.70 (0.64–0.78)	**8.85 (7.01–10.99)**	**5.32 (1.75–11.98)**	**12.27 (9.12–16.02)**	**6.87 (4.40–10.12)**
NLS = 2	41	386	176	11,421	0.58 (0.55–0.60)	18.89 (13.91–24.75)	96.73 (96.39–97.04)	5.78 (4.31–7.74)	0.84 (0.79–0.89)	**9.60 (6.98–12.80)**	**8.00 (2.22–19.23)**	**11.05 (6.88–16.55)**	**10.24 (6.08–15.89)**
PLS = 2	19	159	198	11,648	0.54 (0.52–0.56)	8.76 (5.35–13.34)	98.65 (98.43–98.85)	6.50 (4.12–10.26)	0.92 (0.89–0.96)	**10.67 (6.55–16.17)**	**20.83 (7.13–42.15)**	**12.36 (6.33–21.04)**	**5.36 (1.12–14.87)**

Bold plus italic values represent a PPV of 5% or higher. Bold values represent a PPV of 3% or higher, the threshold above which the National Institute for Health and Care Excellence recommends investigation for cancer. Italic values represent a PPV of 2–3%.

*WL* weight loss, *TP* true positive, *FP* false positive, *FN* false nositive, *TN* true negative, *AUC* area under the curve, *Sens* sensitivity, *Spec* specificity, *PLR* positive likelihood ratio, *NLR* negative likelihood ratio, *y* years, *mGPS* modified Glasgow prognostic score, *NLS* neutrophil–lymphocyte score, *PLS* the platelet–lymphocyte score.

For most age-groups with unexpected WL, the PPV for a single abnormal inflammatory marker or a raised score was above 3%. PPVs for individual abnormalities were lower than when they were combined as a score. For example, the PPV for cancer increased from 7.06% (95% CI 5.43–8.99%) for low albumin and 6.26% (5.34–7.29%) for raised CRP to 8.85% (7.01–10.99%) when both were combined as mGPS = 2. While 2408 fewer people with unexpected WL would be referred unnecessarily per 100,000 people tested, using an mGPS = 2 compared to a raised CRP, 125 fewer people with cancer would be referred for investigation.

## Discussion

### Summary

Our findings support the use of individual inflammatory marker abnormalities, in particular a raised CRP, in preference to inflammatory marker scores to triage people attending primary care with unexpected WL for cancer investigation.

### Strengths and limitations

We ensured a consistent study population by selecting participants with all five inflammatory markers recorded. This strategy is likely to have resulted in an enriched population judged to be at higher risk of cancer. Further research is necessary to establish the generalisability of our findings. The outcome was all stages and types of cancer combined with follow-up limited to the period for which the association between unexpected WL and cancer diagnosis has been reported.^8^ A larger sample size would have permitted stratification by cancer site and stage to inform the investigative approach.

### Previous literature

Without taking symptoms into account, an AUC of 0.64 (95% CI 0.63–0.65), sensitivity of 46.1% (44.0–48.1) and specificity of 75.4% (74.1–75.6%) was reported for a cancer diagnosis within 1 year of a CRP ≥ 7 mg/l in a cohort of 111,440 people tested in primary care.^10^ Despite using a shorter follow-up period and higher CRP threshold, the AUC for CRP in our cohort was higher with increased sensitivity and specificity. Adding hypalbuminaemia to create an mGPS = 2 markedly lowered sensitivity and elevated specificity showing the rule-in potential of paired abnormalities.

### Implications

Single inflammatory marker abnormalities are associated with an increased risk of cancer in people attending primary care with unexpected WL, above the current threshold for investigation used in English guidelines, and especially in people aged ≥60 years. If scores including pairs of abnormal inflammatory markers were instead used to select patients for cancer investigation, fewer people would be investigated to diagnose one cancer, with fewer false positives, but fewer people with cancer would be diagnosed overall. For example, there were 159 true positives for abnormal CRP alone, but 74 true positives if using mGPS = 2 combining raised CRP and low albumin. It is possible that combinations of multiple normal inflammatory markers hold greater potential to rule-out cancer than pairs of normal results. This approach warrants further investigation in clusters of symptomatic patients as it would mirror the reassurance taken from panels of normal tests by GPs and patients in clinical practice.

## Conclusion

In people attending primary care with unexpected weight loss individual inflammatory marker abnormalities, notably a raised CRP, are more sensitive than inflammatory marker scores. The associated risk of cancer is high enough to justify cancer investigation.

## Data Availability

This study is based on CPRD data and is subject to a full licence agreement, which does not permit data sharing outside of the research team.
